# N-Acetyl Cysteine (NAC)-Directed Detoxification of Methacryloxylethyl Cetyl Ammonium Chloride (DMAE-CB)

**DOI:** 10.1371/journal.pone.0135815

**Published:** 2015-08-14

**Authors:** Yang Jiao, Sai Ma, Jing Li, Lequn Shan, Yingjie Wang, Min Tian, Yanwei Yang, Jinlong Sun, Jinghao Ban, Jihua Chen

**Affiliations:** 1 State Key Laboratory of Military Stomatology, Department of Prosthodontics, School of Stomatology, the Fourth Military Medical University, Xi’an, PR China; 2 Department of Orthopaedic Oncology, Xijing Hospital Affiliated to the Fourth Military Medical University, Xi’an, PR China; 3 Department of Orthopaedic Surgery, Tangdu hospital, the Fourth Military Medical University, Xi’an, PR China; 4 State Key Laboratory of Military Stomatology, Department of General and Emergency, School of Stomatology, the Fourth Military Medical University, Xi’an, PR China; University of Pecs Medical School, HUNGARY

## Abstract

Methacryloxylethyl cetyl ammonium chloride (DMAE-CB) is a polymerizable antibacterial monomer and has been proved as an effective strategy to achieve bioactive bonding with reliable bacterial inhibitory effects. However, the toxicity of DMAE-CB may hamper its wide application in clinical situations. Thus, this study was designed to investigate the toxicity of DMAE-CB and explore the possible protective effects of N-acetyl cysteine (NAC). High performance liquid chromatography (HPLC) and liquid chromatography-mass spectrometry (LC-MS) analysis showed that chemical binding of NAC and DMAE-CB occurred in a time dependent manner. Pre-incubation of fourty-eight hours is required for adequate reaction between DMAE-CB and NAC. DMAE-CB reduced human dental pulp cells (hDPCs) viability in a dose-dependent manner. The toxic effects of DMAE-CB were accompanied by increased reactive oxygen species (ROS) level and reduced glutathione (GSH) content. NAC alleviated DMAE-CB-induced oxidative stress. Annexin V/ Propidium Iodide (PI) staining and Hoechst 33342 staining indicated that DMAE-CB induced apoptosis. Collapsed mitochondrial membrane potential (MMP) and activation of caspase-3 were also observed after DMAE-CB treatment. NAC rescued hDPCs from DMAE-CB-induced apoptosis, accompanied by lower level of MMP loss and caspase-3 activity. This study assists to elucidate the mechanism underlying the cytotoxic effects of DMAE-CB and provides theoretical supports for the searching of effective strategies to reduce toxicity of quaternary ammonium dental monomers.

## Introduction

Application of quaternary ammonium monomers, such as 12-methacryloyloxydodecylpyridinium bromide (MDPB)[1] and methacryloxylethyl cetyl ammonium chloride (DMAE-CB)[2, 3], is an effective strategy to achieve dental polymers with antibacterial activities[3–5]. However, the toxic effects of quaternary ammonium monomers may hamper the safe and wide applications of these monomers in clinical situations. Similar to conventional monomers like 2-hydroxy ethyl methacrylate (HEMA) and triethylene glycol dimethacrylate (TEGDMA), the toxic effects of quaternary ammonium monomers have been proved in various cell lines[6–8]. For conventional monomers, much evidence indicates that their cytotoxicity is associated with the disturbed intracellular redox equilibrium[9, 10]. However, whether oxidative stress is involved in quaternary ammonium monomer-related toxicity is still largely unknown. Thus, one of the purposes of the present study was to investigate whether the cytotoxicity of the antibacterial monomer DMAE-CB is accompanied with intracellular oxidative stress.

Tremendous studies indicate that NAC, a well-known antioxidant, is effective in protecting cells from monomer-induced cytotoxicity[11–14]. However, little is known about the effects of NAC on the cytotoxicity of quaternary ammonium monomers. In our preliminary studies, we treated cells with NAC and DMAE-CB mixture and found that NAC did not show remarkable protective effects (unpublished data). This finding is similar to the published data in Ma’s paper[7]. They found that NAC only slightly reduced the cytotoxicity of MDPB when it is mixed with MDPB and used to treat cells immediately. However, when the mixture of MDPB and NAC was incubated with for 24 h before used for cell treatment, NAC remarkably reduced the cytotoxicity of MDPB. Such phenomenon was explained by the time-dependent direct Michael addition reaction between MDPB and NAC which resulted in the reduced levels of free toxic MDPB. Considering that both MDPB and DMAE-CB are quaternary monomers with similar chemical structures, we suppose that DMAE-CB may also form an adduct with NAC through Michael addition reaction. Thus, the second purpose of the present study is to investigate whether NAC could directly react with DMAE-CB, and thus reduce its toxic effects.

## Materials and Methods

### Analysis of DMAE-CB-NAC adduct formation with high performance liquid chromatography (HPLC)

Pure DMAE-CB was added into 10 mM NAC (Sigma; St Louis, MO, USA) solution (in water) to a final concentration of 8 mM. The mixture was neutralized with 1 mM NaOH, and then analyzed immediately after mixing or after 24 h or 48 h of pre-incubation at 37°C. HPLC analysis (Model Shimadzu LC-2010A, Shimadzu Corporation, Kyoto, Japan) was performed at 220 nm on an InertSustain C18 column (5 μm, 4.6 mm × 250 mm, GL Sciences). The column was maintained at 50°C and the samples were eluted for 5 min. The mobile phase, at a flow rate of 0.7 mL/min, consisted of methanol and 0.2% phosphoric acid with 100 mM sodium perchlorate. The volume of sample injected was 20 μL. Identification of the analytes, NAC, DMAE-CB and the possible adduct, was made based on the retention time (RT) of the peaks registered for the standard solutions. The experiment was repeated for three times for each group.

### Analysis of DMAE-CB-NAC adduct with liquid chromatography-mass spectrometry (LC-MS)

The reaction mixture of NAC and DMAE-CB (in water) after pre-incubation of 48 h was further analyzed using LC-MS to confirm the formation of the DMAE-NAC adduct. LC-MS analysis was performed using an Agilent 6460 triple quadrupole MS (Agilent Technologies) equipped with electrospray ionization source. Electrospray ionization (ESI) was performed in negative ion mode. After optimization, the source parameters used were as follows: source temperature was set at 300°C, nebulizer gas (nitrogen) flow rate was 10 L/min, sheath gas temperature was 400°C, sheath gas (nitrogen) flow rate was 6 L/min and the spray voltage was -3500 V. Data were acquired in MS scanning mode and recorded in the 100–800 m/z region. The analysis was repeated for three times for each group. Peak detection, integration and quantitative analysis were done using Mass Hunter Quantitative analysis software (Agilent Technologies).

### Cell cultures

HDPCs were isolated from dental pulp tissue of non-carious third molars extracted from young healthy patients (18–25 years old) according to a protocol approved by the Ethics Committee of the Fourth Military Medical University (approval number: 15–20) with written informed consent obtained from all subjects. Following extraction, the teeth were delivered to the cell culture laboratory in isolation medium containing alpha-modified eagle’s medium (α-MEM; Gibco, Gaithersburg, MD, USA) supplemented with 10% fetal bovine serum (Gibco, Gaithersburg, MD, USA), 100 units/mL penicillin and 100 mg/mL streptomycin. The dental pulp tissue was then minced and digested in a solution containing 3 mg/mL type I collagenase and 4 mg/mL dispase (Gibco, Gaithersburg, MD, USA) at 37°C for 2 h. Single-cell suspension was obtained by passing the cells through a 70-mm strainer (BD Falcon, Franklin Lakes, NJ, USA). The cells were then cultured in α-MEM (Gibco, Gaithersburg, MD, USA) supplemented with 10% fetal bovine serum (Gibco, Gaithersburg, MD, USA), 100 units/mL penicillin and 100 mg/mL streptomycin. The culture medium was changed every 3 days.

To investigate the influence of NAC on DMAE-CB-induced intracellular oxidative stress and apoptosis, cells were treated with DMAE-CB alone or DMAE-CB+NAC before subjected to various assays. NAC, DMAE-CB or the mixture of DMAE-CB and NAC were all dissolved in complete culture medium. According to the results of HPLC and LC-MS, adequate reaction between DMAE and NAC occurred after at least 48 h of pre-incubation. Thus, for the DMAE-CB+NAC group, DMAE-CB and NAC were mixed and pre-incubated for 48 h before cell treatment.

### CCK-8 assay

The viability of hDPCs was determined using the Cell Counting Kit-8 (CCK-8) assay kit (Beyotime Biotechnology, China) according to the manufacturer’s instructions. HDPCs (Passage two) were seeded into 96-well culture plates at a density of 5 × 10^3^ cells/well and incubated at 37°C and 5% CO_2_. When the cells grew to 80% confluence, DMAE-CB with different concentrations was added into the medium. After an incubation period of 24 h, CCK-8 solution was added to the medium and incubated at 37°C for 4 h. The absorbance of the colored solution was measured using a microplate reader (Bio-Rad Laboratories, Hercules, CA) at a test wavelength of 450 nm. Cells in the control group were treated with culture medium without DMAE-CB. The experiment was repeated for three times with six replicates. The results were plotted as means ± SD.

### ROS analysis

The level of intracellular ROS was evaluated using a commercial kit (Beyotime Biotechnology, China). Cells were cultured and treated with NAC, DMAE-CB or DMAE-CB+NAC for 6 h. Then the cells were incubated with serum-free medium containing 2’,7’-Dichlorofluorescin diacetate (DCF-DA) for 20 min at 37°C. After excessive DCF-DA was removed by washing, the cells were harvested and subjected to flow cytometric analysis (Becton-Dickinson FACScan). For each sample, 10000 cells were analyzed. The results were presented as means ± SD of three independent experiments.

### Intracellular GSH analysis

Intracellular levels of GSH and oxidated glutathione (GSSG) were measured using a commercially available kit (Beyotime Biotechnology, China) according to the manufacturer’s protocol. After treatment with NAC, DMAE-CB or DMAE-CB+NAC for 24 h, the cells were collected and resuspended in the buffer for lyticase (Sigma, USA) for 30 min at 30°C. Then the lysate was centrifugated (10000×g, 5 min, 4°C), and the supernatant was collected. Thiol-reactive fluorescent dye 5-chloromethylfluorescein diacetate (CMFDA) was added to the collected supernatant and then the fluorescence of the samples was evaluated using a microplate reader (Bio-Rad Laboratories, Hercules, CA) at 405 nm. Total protein concentrations of parallel samples were measured using a BCA Protein Assay Kit (Beyotime Biotechnology, China). GSH and GSSG concentration was normalized to protein contents. The experiment was done in triplicate and repeated for three times.

### Annexin V/PI staining

Annexin V/PI staining was performed according to the manufacturer’s instructions (Beyotime Biotechnology, China). HDPCs were seeded into a 6-well plate at a density of 1×10^5^ cells/well. When the cells grew to 80% confluence, the cells were treated with NAC, DMAE-CB or DMAE-CB+NAC, respectively. For the control group, cells were treated with only culture medium. After various treatments of 24 h, hDPCs were collected and washed with PBS, gently resuspended in binding buffer, and incubated with Annexin V/PI. Flow cytometry analysis was performed using Cellquest software (FACSCalibur, BD, USA). For each sample, 10000 cells were analyzed. The results were presented as means ± SD of three independent experiments.

### Hoechst 33242 staining

After treatment with NAC, DMAE-CB or DMAE-CB+NAC for 24 h, the cells were stained with Hoechst 33342 (Beyotime Biotechnology, China) for 5 min. Cell morphology was examined under a fluorescent microscope (Olympus, Tokyo, Japan).

### Measurement of mitochondrial membrane potential

The mitochondrial membrane potential (MMP) was assessed using the fluorescent dye JC-1 (Beyotime Biotechnology, China). After treatment with NAC, DMAE-CB or DMAE-CB+NAC for 24 h, the cells were incubated with the JC-1 staining solution (5 μg/ ml) for 20 min at 37°C. After rinsing for two times with JC-1 staining buffer, the fluorescence intensity of JC-1 aggregates was detected at an excitation wavelength of 525 nm and emission wavelength of 590 nm, whereas the JC-1 monomer was measured at an excitation wavelength of 490 nm and emission wavelength of 530 nm using flow cytometric analysis (Becton-Dickinson FACScan). For each sample, 10000 cells were analyzed. The results were presented as means ± SD of three independent experiments.

### Caspase-3 activity

A caspase-3 colorimetric assay kit (Beyotime Biotechnology, China) was used to evaluate caspase-3 activity. After treatment with NAC, DMAE-CB or DMAE-CB+NAC for 24 h, the cells were lysed in lysis buffer. The cell lysates were tested for protease activity using Asp-Glu-Val-Asp-pNA (DEVD-pNA), a tetrapeptide p-nitroanilide substrate. After incubation with this substrate for 2 h, the absorbance was measured at 405 nm using a microplate reader (Bio-Rad Laboratories, Hercules, CA). The experiment was done in triplicate and repeated for three times.

### Statistical analysis

Values were expressed as the mean ± SD and the data were analyzed by one-way ANOVA analysis of variance followed by Tukey’s test for multiple comparisons. The level of significance was set at p ≤ 0.05.

## Results

### Analysis of NAC-DMAE-CB adduct formation by HPLC

**Fig 1:**
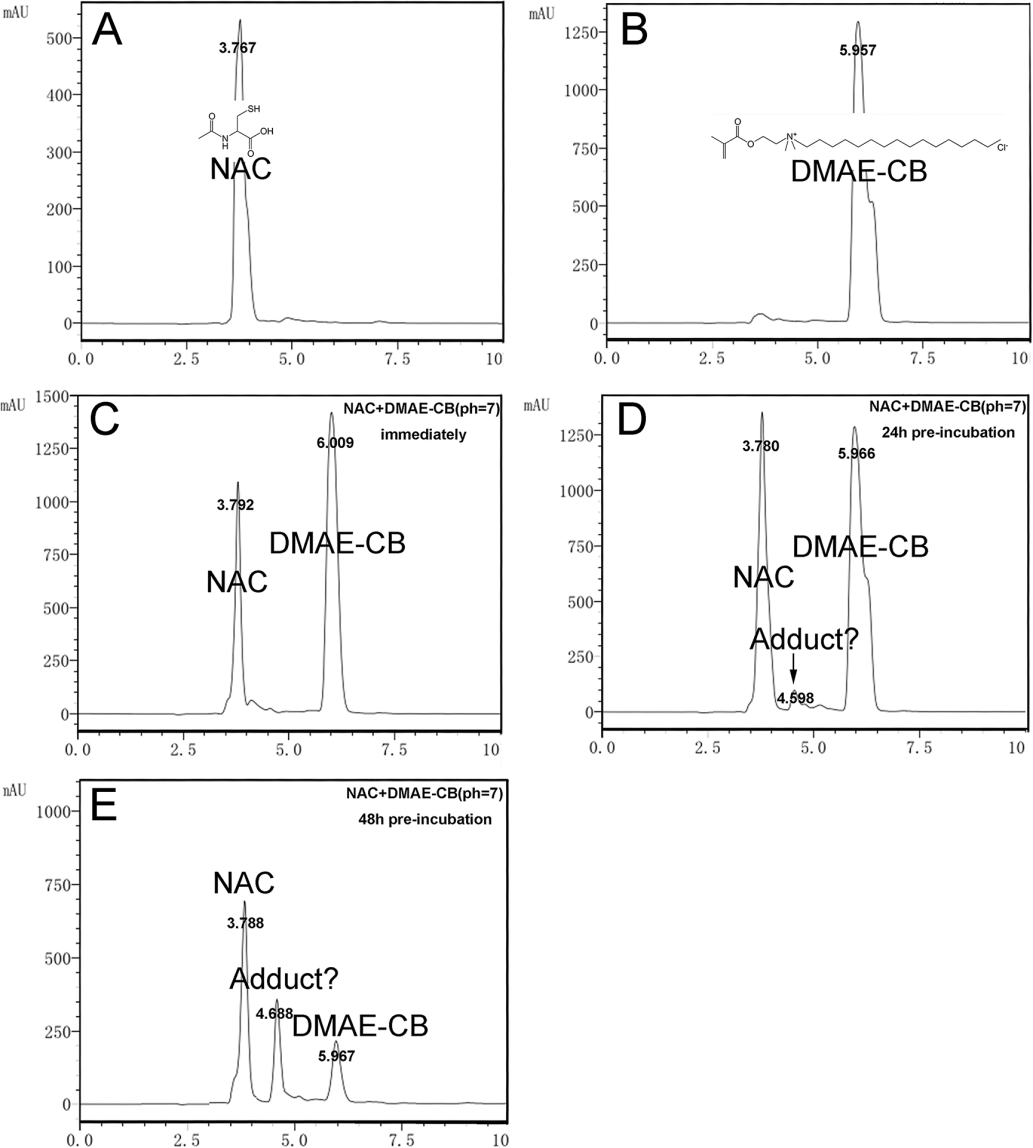
Analysis of the NAC-DMAE-CB adduct using HPLC. NAC (A) or DMAE-CB (B) solution was first analyzed alone. Then, analysis of the mixtures of NAC and DMAE-CB was performed immediately after preparation (C), or after 24 h (D) or 48 h (E) of pre-incubation.

Typical graphs of HPLC analysis are presented in Fig 1. The solution of NAC alone showed a peak at RT of 3.767 min (Fig 1A), and DMAE-CB exhibited a peak at RT of 5.957 min (Fig 1B). For the mixture in which DMAE-CB and NAC were mixed without pre-incubation, no extra peak could be detected (Fig 1C). However, when pre-incubation of 24 h or 48 h was performed before the HPLC study, the NAC/DMAE-CB mixture gave rise to an extra peak at RT of 4.598/4.688 min (Fig 1D and 1E), indicating that a new component was formed during the incubation period. Besides, the amount of the new component increased with prolonged incubation time. The increase of the new component was accompanied by a concomitant decrease of NAC and DMAE-CB.

### Analysis of NAC-DMAE-CB adduct by LC-MS

**Fig 2:**
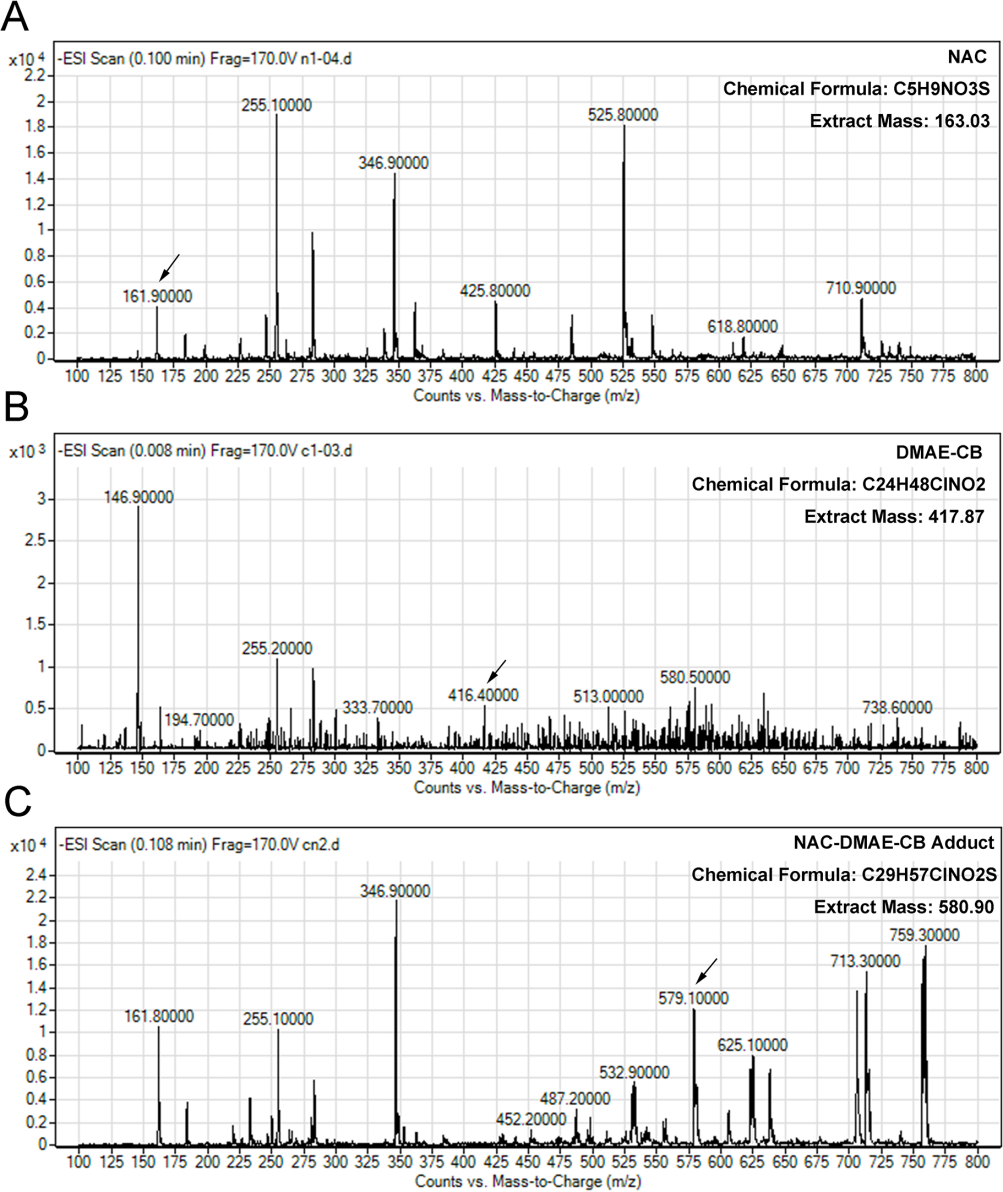
Detection of NAC-DMAE-CB adduct by LC-MS after 48 h of incubation. A, B and C shows the full scan MS spectra of NAC (m/z = 161.9, [M-H]), DMAE-CB adduct (m/z = 416.7, [M-H]) and NAC-DMAE-CB, respectively adduct (m/z = 579.1, [M-H]). Arrows indicate the peak of the three components.

Typical graphs of the LC-MS analysis are presented in Fig 2. The three eluted components (as indicated by the three peaks) were subsequently individually analyzed using mass spectrometry. One of the eluted components gave rise to a peak at m/z 161.9 in the mass spectrum, corresponding to that of protonated NAC (Fig 2A). The peak arising at m/z 416.7 was generated by another component, and this peak can be ascribed to DMAE-CB (M-H) (Fig 2B). As can be seen in Fig 2C, when the third eluted component was analyzed by MS, it showed a peak at m/z = 579.1. This peak was in accordance with a protonated 1:1 adduct of NAC and DMAE-CB (M-H), showing that an adduct of NAC and DMAE-CB was formed.

### Effect of DMAE-CB on cell viability

**Fig 3:**
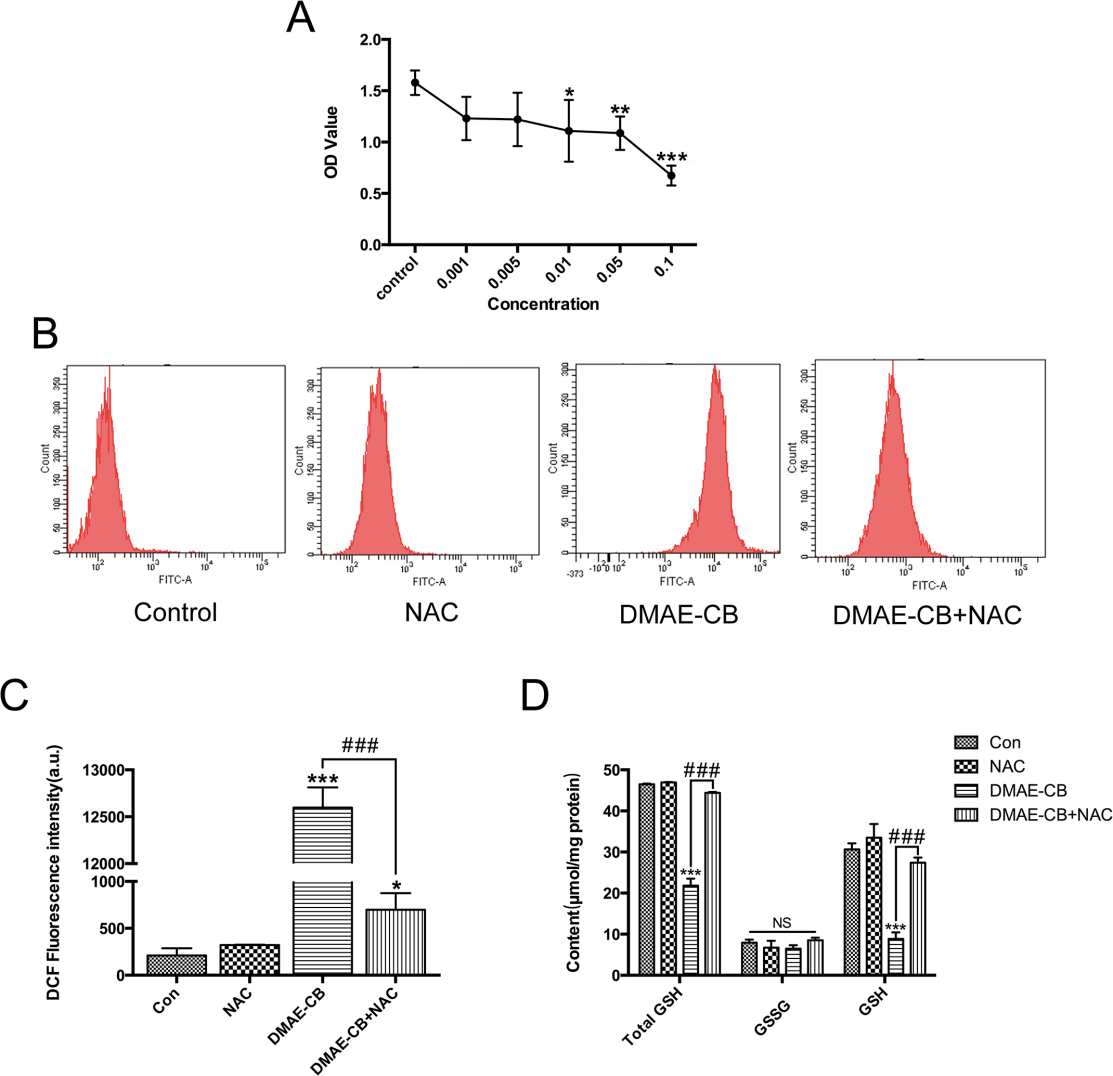
Influence of DMAE-CB on cell viability and oxidative stress and the rescuing effects of NAC. A. Cytotoxicity of DMAE-CB on hDPCs as evaluated by CCK-8 assay (n = 6). B-D. Effects DMAE-CB on intracellular oxidative stress and the rescuing effect of NAC. Intracellular ROS levels (B,C) and contents of GSH (D) in hDPCs were presented, respectively (n = 3). (*p<0.05, **p<0.01, ***p<0.001 versus control group; #p<0.05, ##p<0.01, ###p<0.001 versus DMAE-CB-treated cells; one-way ANOVA).

To examine the influence of DMAE-CB on viability of hDPCs, cells were treated with various concentrations (0.001–0.005–0.01–0.05–0.1 mM) of the monomer for 24 h and then analyzed using CCK-8 assay. As shown in Fig 3A, DMAE-CB induced a dose-dependent decrease in cell viability. Compared to control group, statistically significant reduction of cell viability was detected in groups that were treated with DMAE-CB with concentrations of 0.01 mM or higher. In the following studies to investigate DMAE-CB-induced intracellular oxidative stress and apoptosis and the possible rescuing effect of NAC, we used DMAE-CB at 0.01 mM, which is the lowest concentration that could induce significant reduction of cell viability in the CCK-8 assay.

### Effects DMAE-CB on intracellular oxidative stress and the rescuing effect of NAC

Intracellular level of ROS was studied using DCF-DA staining. As can be seen in Fig 3C, DMAE-CB treatment for 6 h induced a burst in intracellular ROS level (p < 0.001). However, when the mixture of DMAE-CB and NAC was pre-incubated for 48 hours and then used to treat the cells, ROS level was remarkably reduced as compared to cells that were treated with DMAE-CB alone (p < 0.001). However, ROS levels in DMAE-CB+NAC mixture group was still higher than that of control group, in which cells were treated with only culture medium (p < 0.05).

The intracellular levels of GSH and GSSH were evaluated using a commercial kit. As shown in Fig 3D, the level of GSH drastically decreased (p < 0.001) after the cells were treated with DMAE-CB for 24h. As expected, the pre-incubating NAC with DMAE-CB for 48 h before cell treatment rescued monomer-induced GSH depletion. As for GSSG content, no statistically significant difference was found among the groups (p > 0.05).

### Effect of DMAE-CB on apoptosis and the rescuing effect of NAC

**Fig 4:**
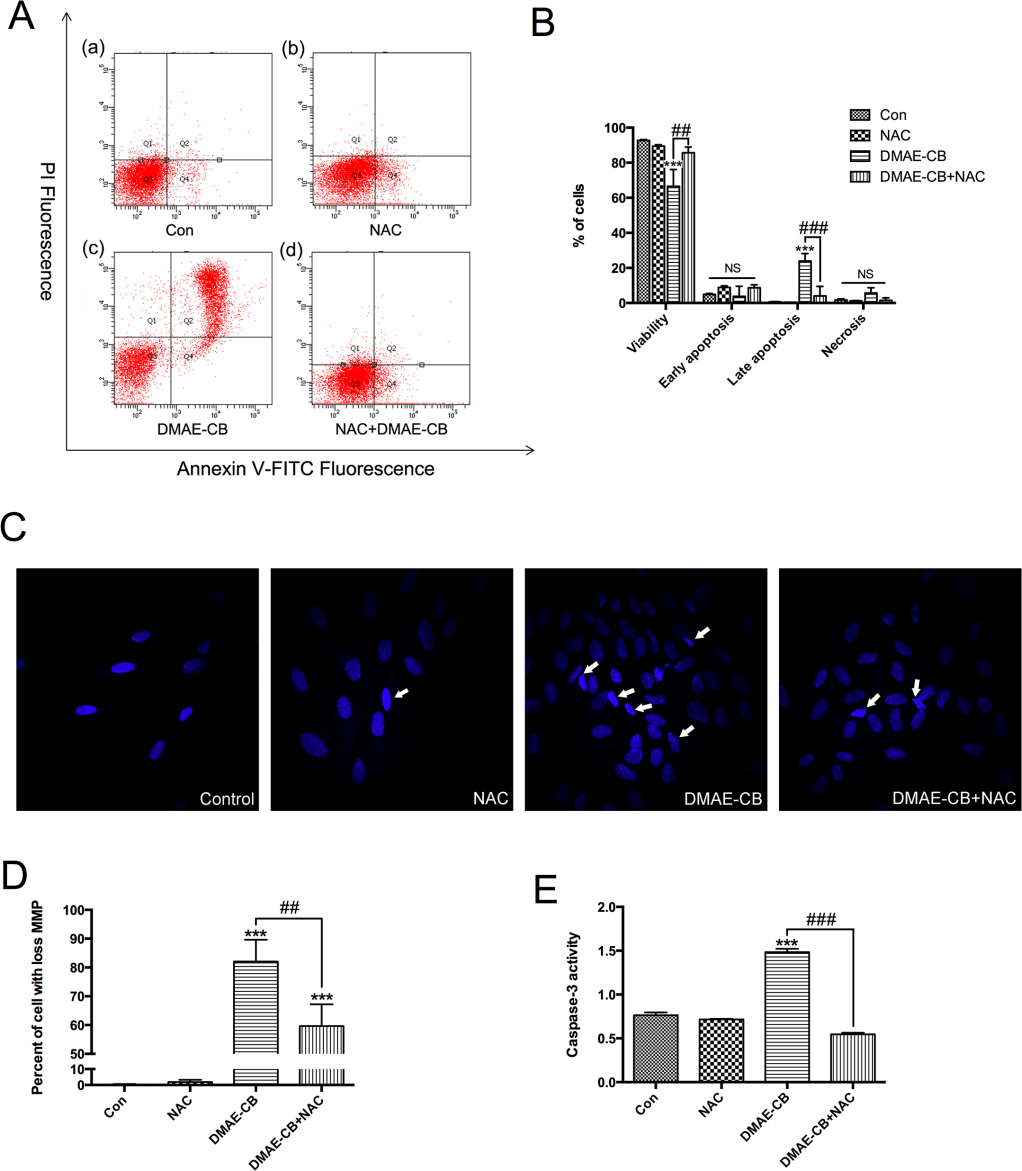
DMAE-CB induced apoptosis and the rescuing effect of NAC. A. Typical graphs of Annexin V/ PI staining for each group. The viable cells (Q3; Annexin V^-^; PI^-^), early apoptosis cells (Q4; Annexin V^+^; PI^-^), late apoptosis cells (Q2; Annexin V^+^; PI^+^) and necrosis cells (Q1; Annexin V^-^; PI^+^) are shown in each quadrant. B, statistical analysis of three independent Annexin V/PI staining analysis. C. Morphological changes of hDPCs. Cells pointed by arrows exhibited typical morphological characteristics of apoptotic cells. D. Mitochondrial membrane potential (MMP). E, activities of caspase3. (*p<0.05, **p<0.01, ***p<0.001 versus control group; #p<0.05, ##p<0.01, ###p<0.001 versus DMAE-CB-treated cells; one-way ANOVA).

To further explore whether apoptosis occurred after DMAE-CB treatment, Annexin V and PI staining by flow cytometry analysis was performed. Treatment with DMAE-CB for 24 h significantly reduced the percent of viable cells (p < 0.001, Fig 4B) as compared to the control group. Meanwhile, the percent of Annexin V (+)/PI (+) late apoptosis cells were remarkably increased (p < 0.001). Furthermore, Hoechst 33342 staining revealed that treatment with DMAE-CB induced cell shrinkage, nuclear condensation and fragmentation, which are typical morphological characteristics of apoptosis (Fig 4C). As can be seen in Fig 4B, when cells were treated with the mixture of DMAE-CB and NAC (with 48-h of pre-incubation), the percent of viable cells was increased and the percent of late apoptosis cells were reduced. This result was in accordance with that of Hoechst staining, which showed a reduced number of cells with typical characteristics of apoptosis.

Since oxidative stress is known to impair mitochondria to trigger the apoptotic process, we investigated the influences of DMAE-CB and DMAE-CB plus NAC on MMP in hDPCs. Cells that loss their MMP can be stained by JC-1 to generate fluorescence. Compared to the control group and the NAC group, higher percent of cells showed loss of MMP after treatment with DMAE-CB for 24 h (p < 0.001, Fig 4D). On the other hand, treatment with pre-incubated DMAE-CB/NAC mixture reduced the percent of cells with collapsed MMP as compared to DAME-CB alone group (p < 0.01).

Caspase-3 is a key downstream effector of apoptosis[15, 16]. DMAE-CB treatment significantly increased caspase-3 activity in hDPLCs (p<0.001, Fig 4E). In contrast, cells treated with pre-incubated mixture of DMAE-CB and NAC showed a significantly reduced activity of caspase-3 as compared to cells treated with DMAE-CB alone (p < 0.001).

## Discussion

In this study, we demonstrate that the antibacterial monomer DMAE-CB induced cell death, especially apoptosis, in hDPCs. DMAE-CB-induced cell death was accompanied with the depletion of GSH and overproduction of ROS. NAC can react with DMAE-CB to form an adduct, and thus reduce the cytotoxic effect of DMAE-CB.

The antibacterial monomer DMAE-CB induced apoptosis in hDPCs. Using the CCK-8 assay, we found that DMAE-CB reduced cell viability in a dose-dependent manner, and the lowest concentration for the monomer to induced significant difference in cell viability was 0.01 mM. To further explore whether apoptosis occured after DMAE-CB treatment, we performed Annexin V/PI staining, Hoechst 33342 staining, JC-1 staining and caspase-3 activity test. Annexin V/PI staining found that after treatment with 0.01 mM of DMAE-CB for 24 h, almost 30% of the tested hDPCs were in the stage of late apoptosis. This result was further confirmed by Hoechst 33342 staining, which revealed that hDPCs displayed typical apoptotic characteristics after DMAE-CB treatment. The loss of mitochondria potential is an important indicator of apoptosis[17], and JC-1 staining found that a large percent of cells showed a collapsed mitochondrial membrane potential after DMAE-CB treatment. Furthermore, DMAE-CB also significantly increased the activity of caspase-3, which is an important effector of apoptosis. These results were in line with our previous study which found that DMAE-CB could activate the intrinsic mitochondria apoptotic in L929 mouse fibroblasts[18].

DMAE-CB-induced apoptosis is related to intracellular oxidative stress. It is well accepted now that dental monomers cause cytotoxic effects by disturbing the intracellular redox balance[10, 13, 19]. Similar to other conventional monomers, we found in this study that DMAE-CB induced a remarkable increase in intracellular ROS level. Furthermore, we found that over-production of ROS was accompanied with a rapid depletion of GSH. More interestingly, the depletion of GSH is not related to an increase in GSSG, which was in accordance with the previous study[20, 21]. This finding indicates that the major mechanism of GSH depletion after DMAE-CB exposure is different from an oxidation of GSH to GSSG. In fact, several studied found that methacrylate monomers could deplete intracellular GSH by covalently bonding with its thiol group[12, 22, 23]. Since GSH is normally involved in reactions that oppose the continuous production of ROS, depletion of GSH by methacrylate monomers could impair this defense and ultimately result in over-production of ROS. These findings indicate that the depletion of GSH by methacrylate monomers, including DMAE-CB, is a key event during the cytotoxic process.

**Fig 5:**
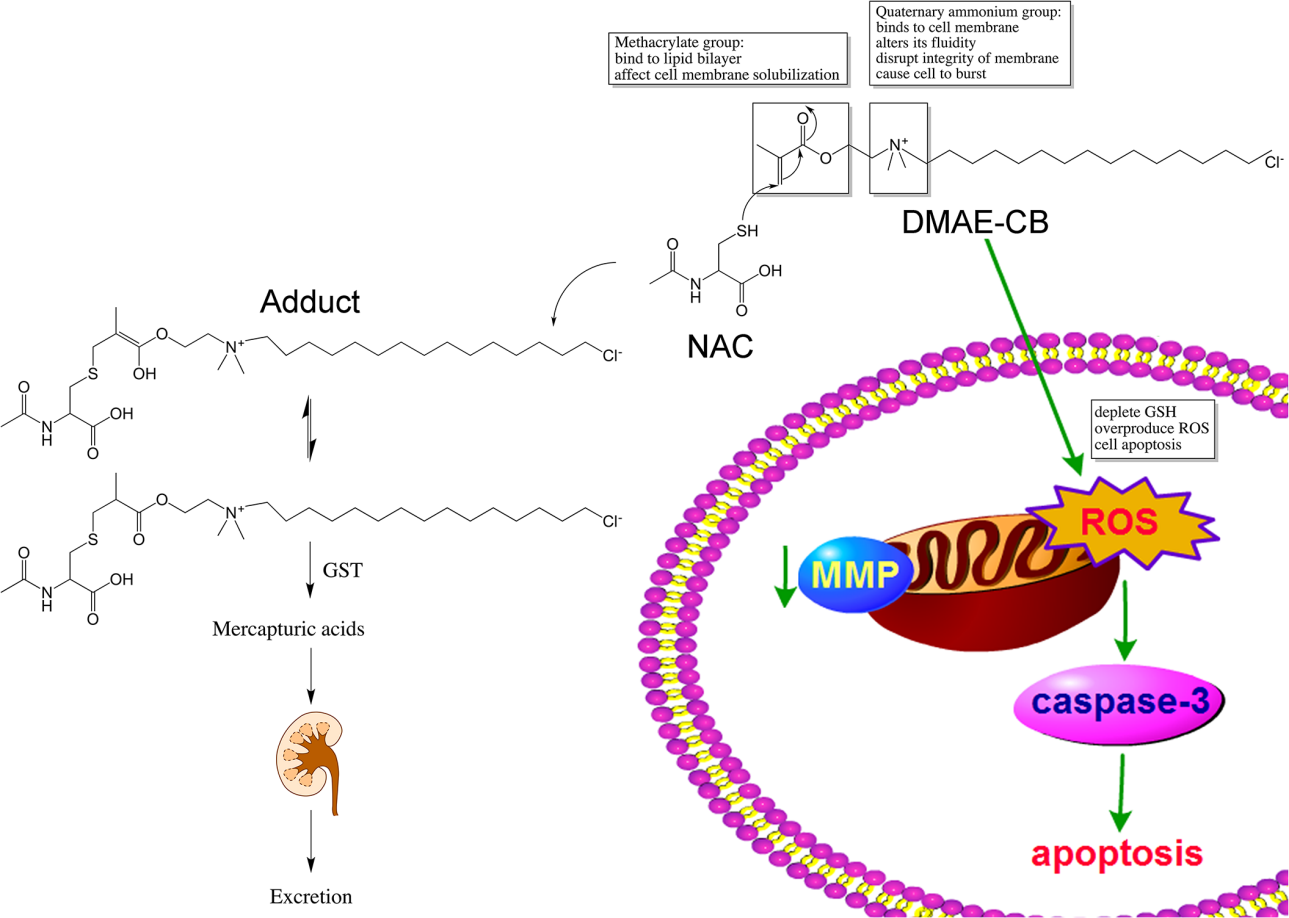
A schematic diagram summarizing the toxicity of the antibacterial monomer DMAE-CB and rescuing effects of NAC. DMAE-CB-induced apoptosis was correlated with the generation of increased ROS level accompanied with reduced GSH content which was followed by the collapsed miltochondrial membrane potential (MMP) and activation of caspase-3. The chemical reaction between NAC and DMAE-CB resulting in the formation of the 1:1 DMAE-CB-NAC adduct through Michael addition reaction, and thus reduce the cytotoxic effect of DMAE-CB. The reaction may result in two isomers, the *enol* form and the *keto* form, and a chemical equilibrium may exist between the two forms. The formed NAC-DMAE-CB adduct are likely to be transformed to mercapturic acids catalyzed by Glutathione S-transferases (GST) and eventually excreted with urine.

Since the depletion of GSH by the methacrylate group of DMAE-CB could be an important mechanism for the toxic effects of the antibacterial monomer, we hypothesized that consumption of the methacrylate group by other molecules may reduce the cytotoxicity of the monomer. Similar to GSH, NAC also has a thiol group, and could covalently bind to the methacrylate group of dental monomers[24–27]. In our previous study, we found that NAC could react with the quaternary ammonium antibacterial monomer MDPB through Michael-type addition reactions, and pre-incubating NAC with MDPB for 24 h could significantly reduce the cytotoxic effects of the monomer[7]. Considering that both MDPB and DMAE-CB are quaternary monomers with similar chemical structures, we suppose that DMAE-CB may also form an adduct with NAC through Michael addition reaction, and thus reduce its toxic effects. To test this hypothesis, first, we performed HPLC analysis. When the mixture of DMAE-CB and NAC was tested after 24 h of incubation, an extra peak, though very small, was observed. When the incubation time was prolonged to 48 h, the extra peak was more pronounced. These results indicate that a new component was formed during the incubation period, and the amount of the new component was increased with prolonged incubation time. To elucidate whether the new component detected in HPLC analysis is the adduct of DMAE-CB and NAC, we further performed LC-MS analysis. Indeed, a peak at m/z = 579.1 was detected, and this peak can be ascribed to the protonated 1:1 adduct of NAC and DMAE-CB (M-H). Altogether, the results of HPLC and LC-MS indicate that chemical reaction between DMAE-CB and NAC could happen, and at least 48 h of incubation is required to allow adequate reaction between the two molecules (Fig 5).

Since HPLC and LC-MS confirmed the reaction between DMAE-CB and NAC, we then performed studied to investigate whether pre-incubating NAC with DMAE-CB could reduce the cytotoxic effects of DMAE-CB. Since HPLC analysis found that at least 48 h of incubation is required to allowed adequate reaction between DMAE-CB and NAC, we pre-incubated the mixture of DMAE-CB and NAC for 48 h before cell treatment. It was found that compared with cells that were treated with DMAE-CB alone, cells treated with pre-incubated NAC/DMAE-CB mixture displayed lower intracellular ROS level and higher GSH level. NAC also rescued the cells from DMAE-CB-induced apoptosis, as indicated by higher percent Annexin V (-)/PI (-) viable cells and lower percent of Annexin V (+)/PI (+) cells. Furthermore, DMAE-CB-induced collapsed MMP and caspase-3 activation was reversed in the NAC/DMAE-CB mixture group. It is possible that the pre-incubation of NAC with DMAE-CB reduced the amount of active methacrylate group and thus protected the cells from DMAE-CB-related cytotoxicity[28]. In addition, the formed NAC-DMAE-CB adduct may be less cytotoxic since they are less reactive, more water-soluble and easy to be cleared from the cells cleared from the cells like conventional monomers[12, 23]. The adducts between NAC and monomers could be transformed to mercapturic acids catalyzed by Glutathione S-transferases (GST) and eventually excreted [26]. Such reaction has been described *in vitro* employing various cell types[29–31] (Fig 5). However, it cannot be ruled out that the protective effect of NAC may also be related to its potent antioxidative effects.

As can be seen from its chemical structure, besides the methacrylate group, the antibacterial monomer DMAE-CB also has a quaternary ammonium group, which is responsible for its bactericidal activity. Since quaternary ammonium compounds kill bacteria by disrupting the integrity of the bacterial cell membrane, we believe that the quaternary ammonium group in DMAE-CB could also solubilize the membrane of hDPCs and thus cause cell death[32, 33]. In other words, the cytotoxicity of the antibacterial monomer is related to both its methacrylate group and quaternary group. NAC may react with DMAE-CB to reduce the toxic effect related to the methacrylate group, but it may not influence quaternary ammonium group-induced disruption of cell membrane. Therefore, while NAC could remarkable reverse the toxic effects of conventional monomer (such as HEMA and TEGDMA)[13, 14], NAC can only partially reduce the toxic effects of DMAE-CB in some critical situation (such as after 48 h of pre-incubation).

## Conclusions

In conclusion, we found in this study that NAC could reduce the cytotoxicity of DMAE-CB when it is pre-incubation with the monomer for 48 h before cell treatment. This detoxification can be partially attributed to the adduct formation resulting from a chemical reaction between DMAE-CB and NAC during the pre-incubation period. The amount of the NAC-DMAE-CB adduct increased with incubation time, suggesting that the chemical reaction between DMAE-CB and NAC occurred in a time-dependent manner. However, whether more amount of the adduct can be produced with even longer pre-incubation time need to be further investigated. Besides, whether reaction between DMAE-CB and NAC would influence the antibacterial activity and the polymerizing activity of the monomer merits further investigation.

## Supporting Information

S1 Data Raw data. (XLS)

S1 Fig Raw data. Graphs of high performance liquid chromatography, liquid chromatography-mass spectrometry and Hoechst 33242 staining.
(PPT)
