# Supplementary figures and images for: N-Acetyl Cysteine (NAC)-Directed Detoxification of Methacryloxylethyl Cetyl Ammonium Chloride (DMAE-CB)
Source: PLoS One. 2015 Aug 14;10(8):e0135815. doi: 10.1371/journal.pone.0135815 (PMC4537128; doi:10.1371/journal.pone.0135815)

## Slide 1
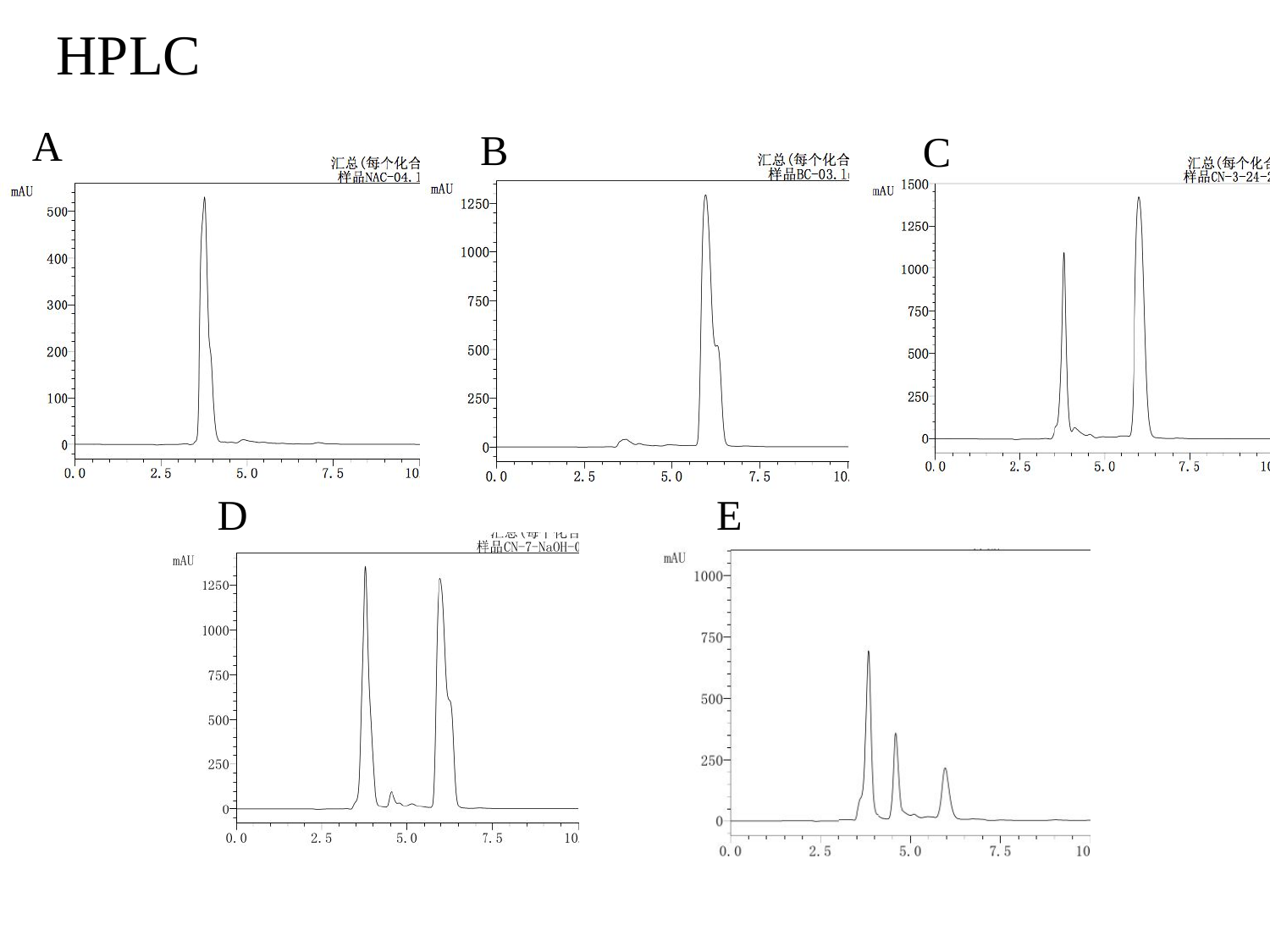

HPLC
A
B
C
D
E

## Slide 2
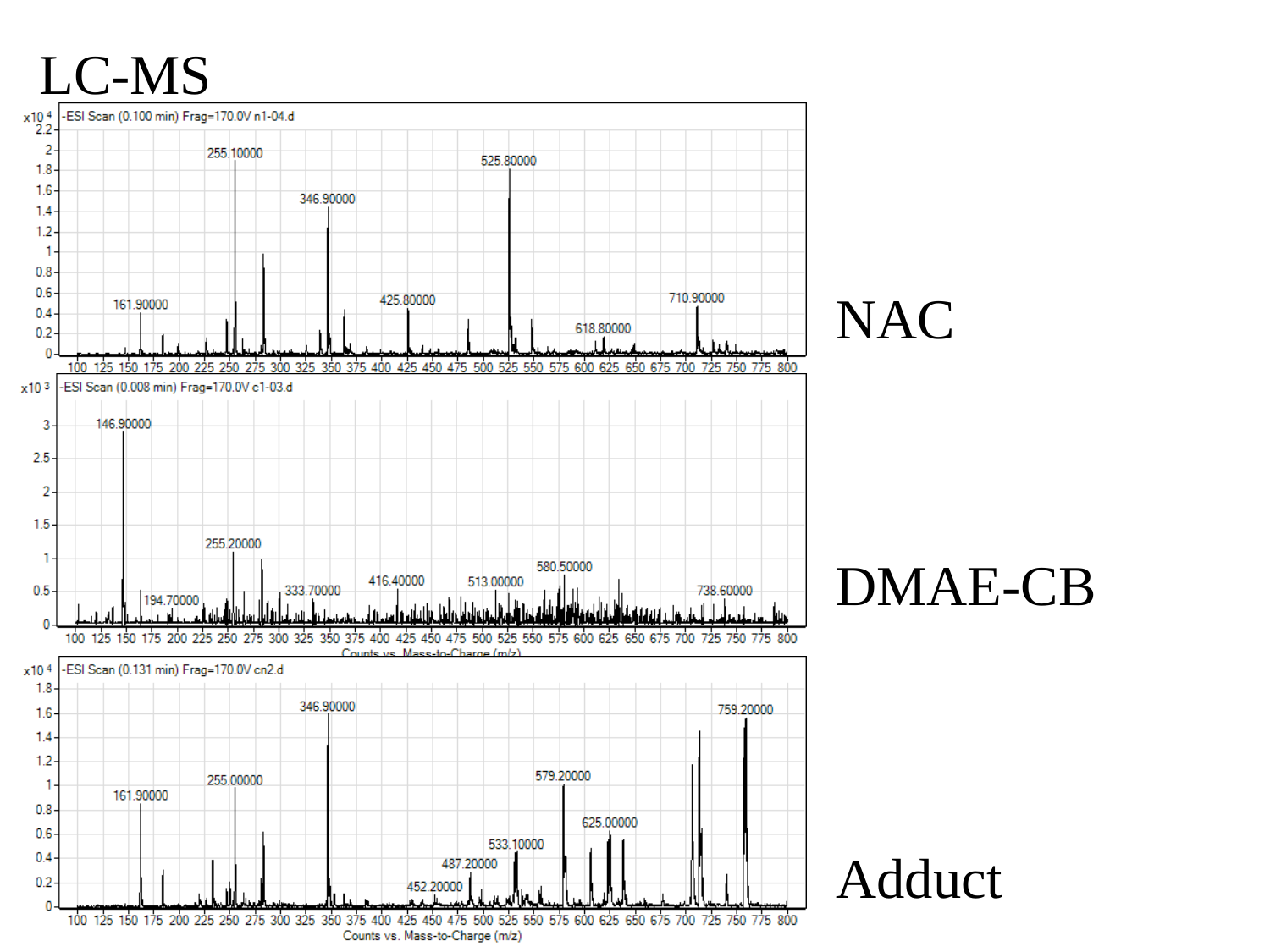

LC-MS
NAC
DMAE-CB
Adduct

## Slide 3
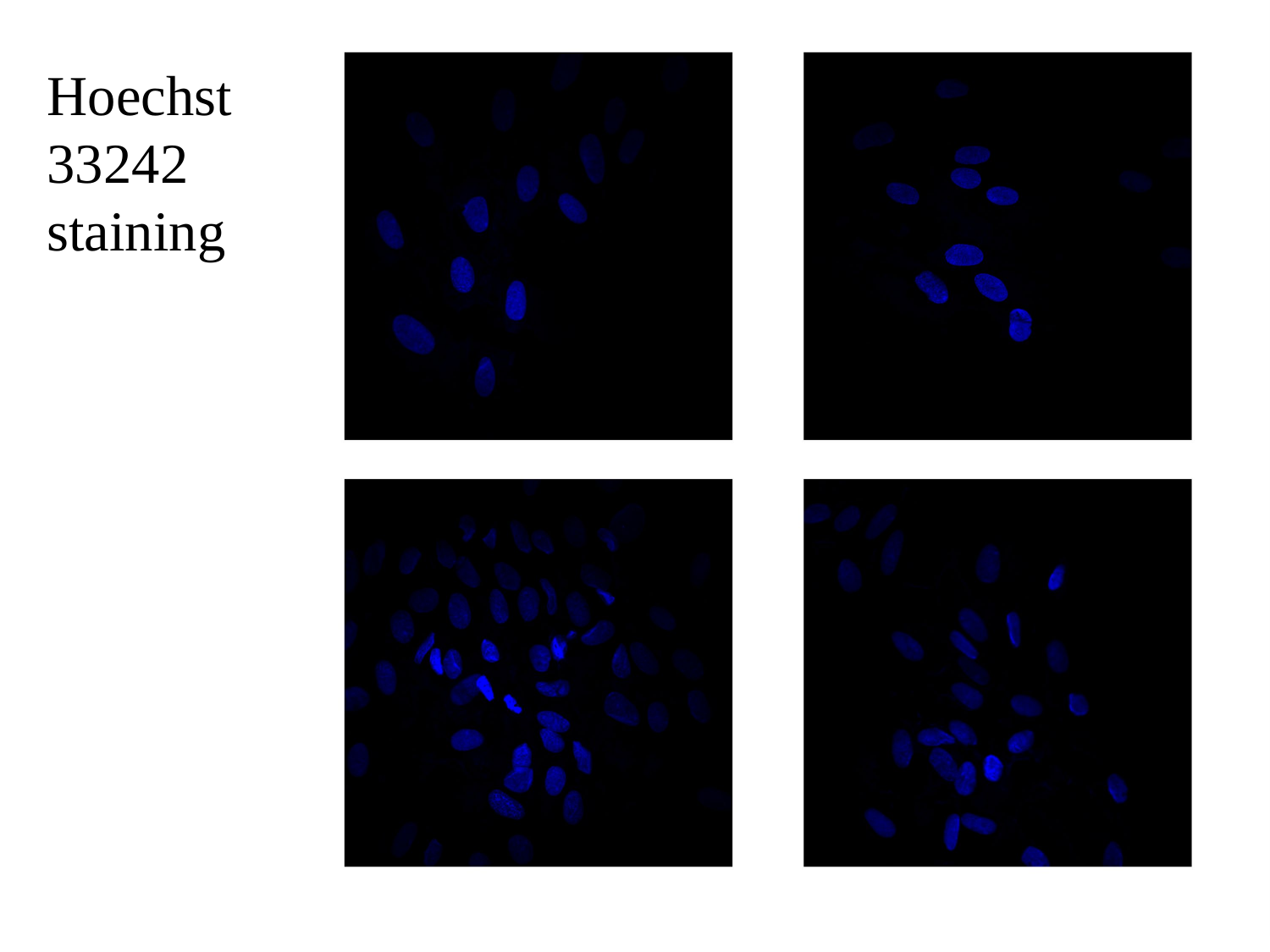

Hoechst
33242
staining

Supplement: S1 Fig — Graphs of high performance liquid chromatography, liquid chromatography-mass spectrometry and Hoechst 33242 staining. (PPT) [file pone.0135815.s002.ppt]
